# Neuroleptic Drugs and PACAP Differentially Affect the mRNA Expression of Genes Encoding PAC1/VPAC Type Receptors

**DOI:** 10.1007/s11064-016-2127-2

**Published:** 2016-11-30

**Authors:** Marta Jóźwiak-Bębenista, Edward Kowalczyk

**Affiliations:** Department of Pharmacology and Toxicology, The Interfaculty Chair of Basic and Clinical Pharmacology and Toxicology, Medical University of Lodz, Zeligowskiego 7/9, 90-752 Lodz, Poland

**Keywords:** Neuropeptides, PACAP, PAC1, VPAC2, Glioma cell line T98G, Neuroleptic drugs

## Abstract

Several lines of evidence suggest that pituitary adenylate cyclase-activating polypeptide (PACAP) is a neuropeptide playing an important role as a neuromodulator. It has been indicated that PACAP is associated with mental diseases, and that regulation of the PACAPergic signals could be a potential target for the treatment of such psychiatric states as schizophrenia. Recent studies have suggested that action of neuroleptic drugs is mediated not only by dopaminergic and serotonergic neurotransmission, but also via neuropeptides which may act both as neurotransmitters and as neuromodulators. The present study examines whether currently-used neuroleptics influence the action of PACAP receptors, whose expression is altered in a schizophrenic patient. Real-time polymerase chain reaction (PCR) was used to examine the effects of haloperidol, olanzapine and amisulpride on the expression of genes coding PAC1/VPAC type receptors in the T98G glioblastoma cell line, as an example of an in vitro model of glial cells. PAC1 mRNA expression fell after 24-h incubation with haloperidol or olanzapine; however the effect was not maintained after 72 h, and haloperidol even up-regulated PAC1 mRNA expression in a dose-dependent manner. All the examined drugs decreased VPAC2 mRNA expression, especially after 72-h incubation. Haloperidol (typical neuroleptic) was distinctly more potent than atypical neuroleptic drugs (olanzapine and amisulpride). In addition, PACAP increased PAC1 and VPAC2 mRNA expression. In conclusion, our findings suggest PACAP receptors may be involved in the mechanism of typical and atypical neuroleptic drugs.

## Introduction

Schizophrenia is characterized by changes in structural network connectivity, which are associated with the nerve cell bodies (synapses, dendrites and axons) as well as with the glial cells. Many studies have shown that structural and functional abnormalities in all three types of glial cells influence the pathogenesis of schizophrenia. There is evidence for reduced numbers of oligodendrocytes, impaired cell maturation and altered expression of astrocyte-related genes, such as reduced expression of GFAP mRNA or glutamine synthetase in schizophrenic patients [1, 2]. Due to the important role of glial cells in the pathology of schizophrenia, efforts to develop glia-directed therapies for the treatment of schizophrenia have been discussed.

Existing antipsychotic drugs improve the negative and positive symptoms of schizophrenia through complex mechanisms that are not completely understood [3, 4]. It is known that they act through the modulation of monoaminergic neurotransmission, primarily involving the dopaminergic pathways [5]. However, studies indicate that antipsychotic drugs used in clinical practice, exert a positive influence on the astrocyte metabolism, which is disturbed in schizophrenia, by binding to astrocyte dopamine receptors and interacting with a wide range of cell compounds [6]. Recent studies have suggested that the neuroprotective action of neuroleptic drugs is mediated by their regulatory influence on both neurotrophic factors and neuropeptides, which may act both as neurotransmitters and as neuromodulators [7–9]. The prominent one among the neuropeptides, which antipsychotics can affect is pituitary adenylate cyclase-activating polypeptide (PACAP) [10]. Nowadays, the signaling system of PACAP is extensively studied due to its pleiotropic involvement in various physiological and pathological conditions.

PACAP is a member of a structurally-related family of peptides whose numbers include vasoactive intestinal peptide (VIP), secretin, glucagon, growth hormone-releasing hormone (GHRH) and peptide histidine–isoleucine (PHI), all being involved in the modulation of numerous biological functions in vertebrates [11]. PACAP is implicated in such phenomena as neurotransmission, neural plasticity, and neurotrophy. It is also endowed with neuroprotective potential [12, 13]. The biological effects of PACAP are mediated by three types of G-protein- coupled receptors (GPCRs), namely PAC1 type (including at least eight different splice variants) and VPAC type (including VPAC1 and VPAC2 subtypes). PAC1 receptors display higher affinity for PACAP than for VIP, while VPAC type receptors bind both peptides similarly and with high affinity [14]. The adenylyl cyclase (AC)/cyclic AMP (cAMP) is the main intracellular signal transduction pathway coupled with all mentioned types of receptors and its dysfunction has been implicated in schizophrenia [15–17].

PACAP and its receptors have been observed to display abnormal activity in a number of psychiatric disorders [14]. The PACAP gene is located on 18p11. Various studies suggest the existence of a link between this locus and schizophrenia [18]. Genetic variants of the genes encoding PACAP and PAC1 have been described in schizophrenic patients [19]. Moreover, the results of recent genetic studies indicate increased VPAC2 receptor expression in schizophrenia [20, 21]. These pharmacological studies suggest that the regulation of PACAP signaling may be a potential therapeutic target for the development of novel antipsychotic drugs [19].

Therefore, the goal of the current paper was to determine the involvement of PACAP in the therapeutic mechanism of neuroleptic drugs, known to be complex, in cells of nonneuronal origin. The study examines the effect of these drugs on the expression of genes coding PAC1/VPAC type receptors in the T98G glioblastoma cell line, as an example of an in vitro model of glial cells, with a typical drug being haloperidol (dopamine D_2_—receptor antagonist), and two atypical drugs being olanzapine (serotonin 5-HT_2A_—receptor antagonist and weak dopamine D_2_—receptor antagonist) and amisulpride (assigned to the group of atypical neuroleptic drugs but it exhibits poor affinity for the serotonin 5-HT_2A_—receptor). Additionally, for the purposes of comparison, the effect of exogenous PACAP38 on its receptors was also evaluated.

## Materials and Methods

*T98G glioma cell line* was purchased from the American Type Culture Collection (ATCC; Rockville, MD, USA). The cells were cultured in 25-ml flasks in medium composed of Advanced MEM supplemented with 10% fetal bovine serum, 2 mM glutamine and a penicillin–streptomycin solution, in a humidified atmosphere of 95% air and 5% CO_2_ at 37 °C. For subcultures, cells were harvested every third day in trypsin-EDTA (0.25% trypsin, 1 mM EDTA) solution. For the gene expression assay, T98G cells were plated onto six-well plates (Nunc) at a density of 3 × 10^5^ cells per well. For the cell viability assay, the cells were seeded onto 96-well plates, at the density of 5 × 10^4^ cells per well. The following substances were used: fetal bovine serum, penicillin–streptomycin solution (5000 units/ml penicillin and 5000 g/ml streptomycin sulphate in normal saline), phosphate buffered saline (PBS; pH 7.4) and trypsin–EDTA were purchased from Invitrogen (Carlsbad, CA, USA). Advanced MEM was obtained from Gibco (Paisley, Scotland, UK).

### Drug and Peptide Treatments

T98G cells were treated with the neuroleptic drugs haloperidole, clozapine, amisulpride (Sigma-Aldrich, St. Louis, Mo., USA), and with peptide PACAP38 (H-8430, Bachem) and incubated for 24 and 72 h. All the neuroleptic drugs were initially dissolved in DMSO followed by a preparation of working concentrations in an appropriate medium. Control samples were treated with medium with DMSO in an amount corresponding to the concentration of the used drugs. The medium was changed every 2 days prior to drug/peptide treatment.

### MTT Conversion

The evaluation of cell viability was performed using the MTT conversion method. Mitochondrial succinate–tetrazolium reductase system converts yellow tetrazolium MTT (3-(4,5-dimethylthiazol-2-yl)-2,5-diphenyltetrazolium bromide, Sigma-Aldrich) into purple formazan. T98G cells were subjected to 24 and 72-h incubation with neuroleptic drugs or without the tested chemicals (control group). After incubation, 50 µl MTT (1 mg/ml) was added and the plates were incubated at 37 °C for 3 h. At the end of the experiment, the cells were exposed to 100 µl dimethyl sulphoxide, which enabled the dissolution of formazan. The absorbance at 570 nm was read on a microplate reader and the results were expressed as a percentage of the absorbance measured in control cells.

### Total RNA Extraction and cDNA Generation

Total RNA extractions were carried out using RNeasy Mini kit (Qiagen) according to the manufacturer’s instructions. RNA content was measured using a PicoDrop spectrophotometer (Picodrop Limited). The quality of RNA samples was analyzed by measuring the absorption ratio at 260/280 nm. The purified total RNA was immediately used for cDNA synthesis or stored at −80 °C.

cDNA was generated with QuantiTect Reverse Transcription Kit (Qiagen) according to the manufacturer’s protocol. Samples of 1 μg of total RNA were used as starting material, reverse transcription was performed in the conditions optimized for use with the kit (42 °C for 30 min, 95 °C for 3 min). The cDNA samples were kept frozen at −20 °C.

### Real Time PCR

Gene expression was measured using the TaqMan kit. Briefly, the reactions were performed in 10 μl amounts including 50 ng cDNA, 5 μl KAPA PROBE FAST qPCR Kit Master Mix ABI Prism (Kapa Biosystems) and 0.5 μl appropriate TaqMan Gene Expression Assay (20×). Specific Pre-made TaqMan assays were used in this study: adenylate cyclase activating polypeptide 1 (pituitary) receptor type I (ADCYAP1R1, Applied Biosystems code-Hs01027974_m1), vasoactive intestinal peptide receptor 2 (VIPR2, Hs00173643_m1), and beta actin (ACTB, Hs01060665_g1) as the endogenous control. TaqMan PCR assays were performed on a 7900HT Fast Real-Time PCR System (Applied Biosystems) in FastGene Fast 96 well PCR plates (Nippon Genetics Europe GmbH). The following thermal cycling specifications were performed: 20 s at 95 °C and 40 cycles each for 3 s at 95 °C and 30 s at 60 °C. Real-time PCR data was analyzed using the 2^−ΔΔCt^ method [22].

### Assay of cAMP Formation

The formation of [3H]cAMP was measured in [3H]adenine prelabeled T98G cells as previously described in detail by Jozwiak-Bebenista et al. [23] and Nowak et al. [24].

### Data Analysis

The results were expressed as the mean ± standard error of the mean (SEM) values and were analyzed for statistical significance using one-way ANOVA, followed by the post hoc Student-Newman-Keul’s test. All parameters were considered significantly different if *p* < 0.05. The statistical analysis was performed using Statgraphics 5.0 plus software (STSC Inc., Rockville, MD, USA).

## Results

### Effects of Neuroleptics on the Viability of T98G Cells

**Fig. 1:**
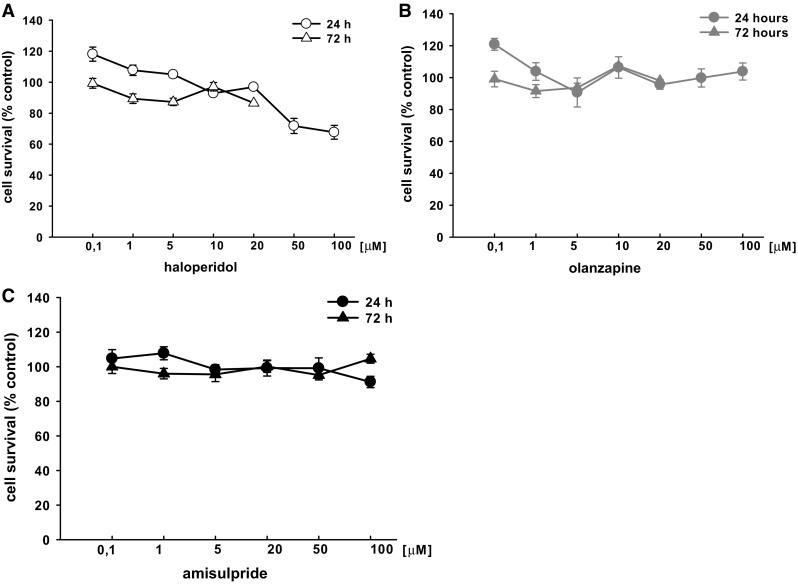
Concentration-dependent effect of neuroleptics (**a** haloperidol, **b** olanzapine, **c** amisulpride) on viability of T98G cells. After 24 and 72 h incubation with the neuroleptic drugs (0.1–100 µM) cell viability was analyzed by MTT test. The results are expressed as percentage of control. Values are the mean ± SEM (n = 6–9)

In order to assess the effects of neuroleptics on cell viability, the cells were seeded on 96-well plates and treated with various concentrations of drugs or vehicle for 24 or 72 h. Following the incubation period, cell viability was measured (Fig. 1). The results revealed that the 24-h treatment with the highest concentrations of haloperidol (50 and 100 µM) caused a marked reduction of cell viability (72% and 68% compared to control value, respectively). Neither the 24-h nor 72-h incubation of cells treated with 0.1–100 μM olanzapine had any effect on cell viability relative to control cells. Indeed, on the basis of the results of the present and comparison studies, it was decided not to evaluate the highest concentrations of haloperidol or olanzapine (50 and 100 µM) on gene expression. Similar to olanzapine, amisulpride was not found to affect cell viability for either incubation time when used at concentrations ranging from 0.1 to 100 μM.

### Effects of Neuroleptics on PAC1 Expression in T98G Cells

The effect of haloperidol, olanzapine and amisulpride on PACAP specific receptors PAC1 gene expression was evaluated in a T98G glioblastoma cell line. The cells were incubated with 5 or 20 µM haloperidol or olanzapine and with 20 or 100 µM amisulpride for 24 or 72 h. After the incubation period, total RNA was extracted and subjected to gene expression studies.

**Fig. 2:**
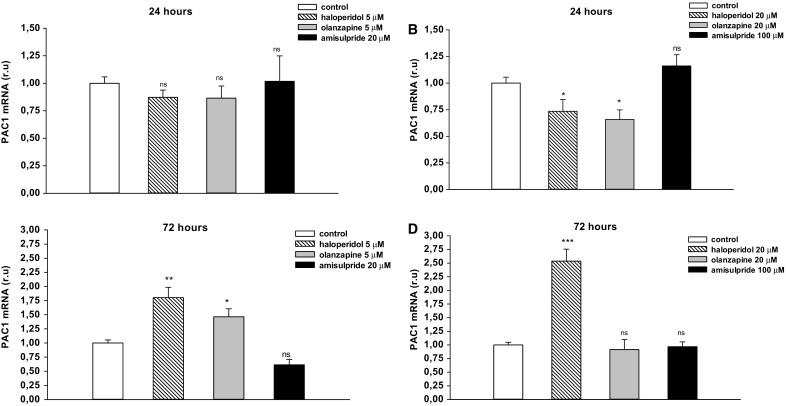
The effects of haloperidol (5 and 20 µM), olanzapine (5 and 20 µM) and amisulpride (20 and 100 µM) on PAC1 expression in T98G cell line. Cells were incubated with neuroleptic drugs for 24 h (**a, b**) or 72 h (**c, d**) and subjected to gene expression studies. Control cells were treated with medium. 1 µg of total RNA isolated from cells was reverse transcribed and 50 ng of cDNA was used for the real-time PCR analysis. All real-time PCR reactions were performed in duplicate, using ACTB gene as an endogenous normalization factor. Results are expressed as relative units (r.u.)—a number resulting from the normalization procedure; values are the mean ± SEM (n = 8). Statistical differences are shown as *p < 0.05, **p < 0.01 and ***p < 0.001

The 24-h incubation with 20 µM haloperidol and olanzapine caused a statistically significant reduction of PAC1 mRNA expression (Fig. 2a, b; *p < 0.05). The 72-h incubation resulted in the up-regulation of PAC1 mRNA expression by haloperidol in a dose dependent manner. Haloperidol administered at 5 µM increased the level of PAC1 mRNA expression by 1.8 times and 20 µM by as much as 2.5 times (Fig. 2c, d; **p < 0.01 and ***p < 0.001). Likewise, although 5 µM olanzapine caused a similar increase in PAC1 expression to haloperidol, no change in PAC1 mRNA expression was revealed at the higher concentration (20 µM) (Fig. 2c, d; *p < 0.05). No statistically significant effect was found for 20 and 100 µM amisulpride on PAC1 expression after either 24-h or 72-h incubation (Fig. 2).

### Effects of Neuroleptics on VPAC2 Expression in T98G Cells

To determine the effect of haloperidol, olanzapine and amisulpride on PACAP-specific receptors, VPAC2 gene expression was evaluated in a T98G glioblastoma cell line. The cells were incubated with 5 and 20 µM haloperidol or olanzapine, or with 20 or 100 µM amisulpride for 24 or 72 h. After the incubation period, total RNA was extracted and subjected to gene expression studies.

**Fig. 3:**
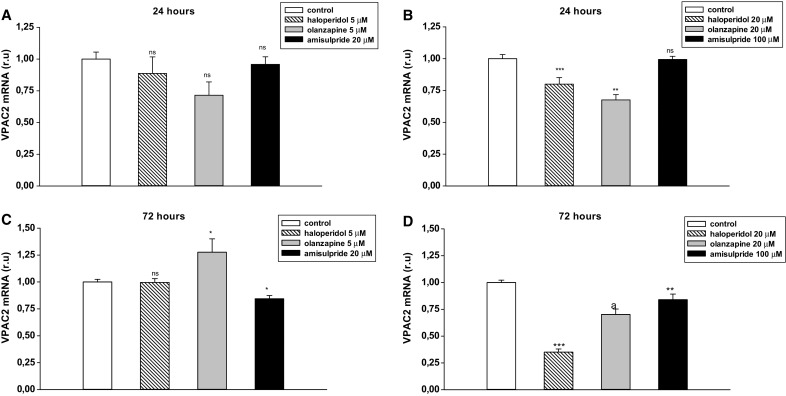
The effects of haloperidol (5 and 20 µM), olanzapine (5 and 20 µM) and amisulpride (20 and 100 µM) on VPAC2 expression in T98 cell line. Cells were incubated with neuroleptic drugs for 24 h (**a, b**) or 72 h (**c, d**) and subjected to gene expression studies. Control cells were treated with medium. 1 µg of total RNA isolated from cells was reverse transcribed and 50 ng of cDNA was used for the real-time PCR analysis. All real-time PCR reactions were performed in duplicate, using ACTB gene as an endogenous normalization factor. Results are expressed as relative units (r.u.)—a number resulting from the normalization procedure; values are the mean ± SEM (n = 8). Statistical differences are shown as *p < 0.05, **p < 0.01 and ***p < 0.001

The 24-h incubation with 20 µM haloperidol and olanzapine caused a statistically significant reduction of VPAC2 mRNA expression (Fig. 3a, b; ***p < 0.001 and **p < 0.01). After a 72-h incubation, the observed diminished effect was maintained by 20 µM olanzapine and further decreased by 20 µM haloperidol (Fig. 3d; ***p < 0.001); however, no effect was observed with the lower concentration of haloperidol. Interestingly, 5 µM olanzapine exhibited the opposite effect to higher concentration of the drug, causing slight up-regulation of VPAC2 (Fig. 3c; *p < 0.05).

The 24-h incubation with amisulpride had no effect on VPAC2 expression. After 72 h, 20 and 100 µM amisulpride caused a weak reduction in VPAC2 mRNA expression (Fig. 3).

### Effect of PACAP38 on PAC1 and VPAC2 Expression in T98G Cells

In parallel, the effect of exogenous PACAP38 on its specific receptors PAC1 and VPAC2 gene expression was evaluated in this cell model. The T98G cells were treated with 1 µM PACAP38 for 24 or 72 h.

**Fig. 4:**
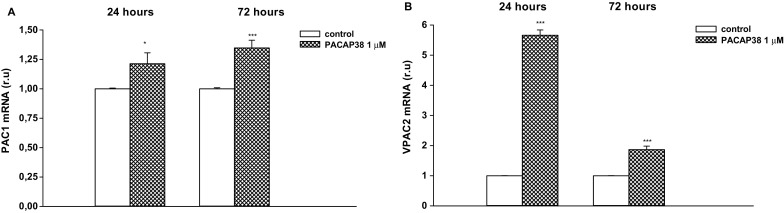
The effects of PACAP38 on PAC1 (**a**) and VPAC2 (**b**) expression in T98 cell line. Cells were incubated with PACAP38 for 24 h or 72 h and subjected to gene expression studies. Control cells were treated with medium. 1 µg of total RNA isolated from cells was reverse transcribed and 50 ng of cDNA was used for the real-time PCR analysis. All real-time PCR reactions were performed in duplicate, using ACTB gene as an endogenous normalization factor. Results are expressed as relative units (r.u.)—a number resulting from the normalization procedure; values are the mean ± SEM (n = 8). Statistical differences are shown as *p < 0.05 and ***p < 0.001

For both incubation times, PACAP38 significantly up-regulated PAC1 and VPAC2 mRNA expression in T98G cells (Fig. 4a, b; *p < 0.05 and ***p < 0.001). PACAP38 was found to have its strongest stimulatory effect on VPAC2 mRNA expression. After a 24-h incubation with peptide, a five-fold up-regulation of VPAC2 mRNA expression was observed compared to the control group (Fig. 4b; ***p < 0.001).

### Effect of PACAP38 and Forskolin on Cyclic AMP Formation in T98G Cells

**Fig. 5:**
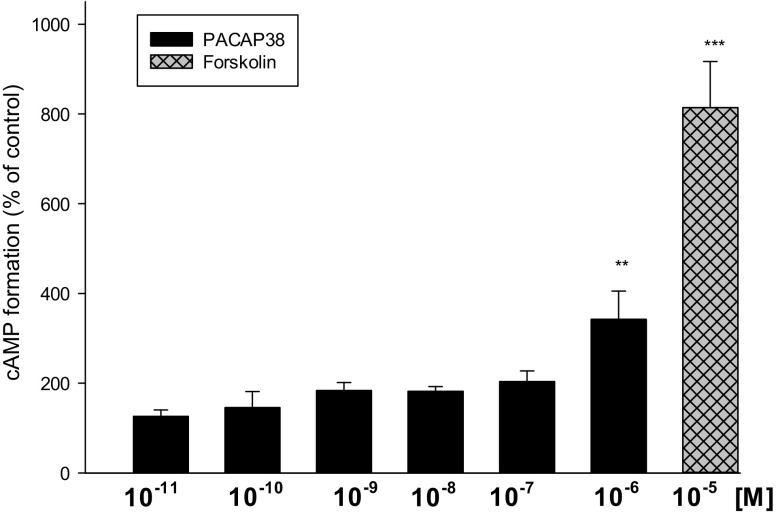
Effects of PACAP38 and forskolin (a direct activator of AC) on cyclic AMP formation in T98G cells. Results are expressed as the mean ± SEM (n = 3–13) and show percent of respective control value. Statistical differences are shown as **p < 0.01 and ***p < 0.001

As shown in Fig. 5, PACAP38 stimulated cAMP production in T98G cells in a dose-dependent manner, reaching a maximal effect at 1 µM. Forskolin, a diterpene derivative acting directly on the catalytic domain of AC was used as reference.

## Discussion

Neuroleptic drugs are well-known for their therapeutic efficacy in schizophrenia, but their exact mechanism of action is still disputed. Although it is known that they alter dopaminergic and serotonergic neurotransmission, the molecular mechanisms underlying the clinical efficacy of typical and atypical antipsychotics in schizophrenia are largely unknown. However, their influence on neurotrophic factors is intriguing. Numerous studies have examined the short- and long-term effects of antipsychotics on the synthesis and secretion of such neuropeptides as nerve growth factor (NGF), brain-derived neurotrophic factor (BDNF) or glial cell line–derived neurotrophic factor (GDNF), which are very important neuroregulators in the etiology and treatment of schizophrenia [8, 25, 26].

However, few reports have examined the effect of neuroleptics on the PACAP signaling pathway. Hyperlocomotion and jumping behavior were attenuated effectively by haloperidol (a D2 antagonist) in PACAP deficient mice, but not deficits in prepulse inhibition (PPI) [27, 28]. Risperidone, a combined D2 and 5-HT_2A_ receptor antagonist, reversed both hyperactivity and diminished PPI in PACAP^−/−^ mice to the level observed in wild-type mice [19]. These studies indicate the presence of an interaction between the PACAP system and the dopaminergic and serotoninergic systems. In addition, it has been clearly confirmed that PACAP may play an important role in psychological disease, and that the regulation of PACAPergic signals could represent a potential target for the pharmacological treatment of such psychiatric states as schizophrenia or bipolar disease [10, 19, 29]. However, before these new drugs enter clinical practice, it is important to determine how existing neuroleptics affect the activity of the PACAPergic pathway. As PACAP and its receptors are known to be involved in the mechanism of antidepressant action [30], the aim of this work was to investigate the effect of neuroleptic drugs used in clinical practice on the expression of genes coding PACAP and VIP peptides and their receptors in the nerve cells.

Three antipsychotic drugs with different mechanisms of action were chosen: a typical mechanism, represented by haloperidol, an atypical one, represented by olanzapine, and amisulpride. The T98G cells were exposed to 5 and 20 µM concentrations of the tested drugs and incubated for 24 or 72 h. These concentrations were chosen based on the literature and our own cell viability measurements (Fig. 1) [31, 32]. Although these concentrations may seem high compared to the therapeutic plasma levels, they are similar to the concentrations of antipsychotic drugs (1–25 µM) that cause GDNF release from C6 cells [25] and to the concentrations of antidepressants (1–10 µM) that alter PACAP mRNA receptors in rat primary cortical neurons [30]. Human glioblastoma cells are used as an example of glial cells in in vitro studies because of their functional similarity to normal astrocytes [7, 33, 34]. Our findings indicate that PACAP38 dose-dependently increased AC activity in both T98G cell membranes and astrocytes (Fig. 5) [23]. Previous studies have also used brain tumor cells to study the regulation of PACAP gene expression [35–38].

The present paper is the first to demonstrate of the expression of PAC1- and VPAC2-subtype PACAP receptors in the T98G human glioblastoma cell line. The expression of the PAC1 and VPAC2 receptors in this type of cells was surprising, taking into account literature data. In contrast, a previous rt-PCR examination of the mRNA of T98G cells only identified the presence of the VPAC2 mRNA receptor, which may be attributed to their use of a less sensitive method [35]. Therefore, the T98G cell line was selected as a suitable model for the study of the action of neuroleptic drugs or exogenous PACAP on PAC1/VPAC-type receptor gene expression.

The treatment of schizophrenia is difficult because of the complex and not fully understood etiology of the disease, although the dopamine, serotonin and glutamate hypotheses are the best known [39, 40]. Another pathophysiological phenomenon associated with schizophrenia is the abnormality in PACAPergic signals, which is present upstream of the regulation of dopaminergic, serotoninergic and glutamergic systems. The hypofunction of the PACAP system leads to the activation of the dopaminergic and serotonergic systems, and the inactivation of the glutamergic system [10]. Therefore, a potentially valuable target for schizophrenia treatment could be the regulation of PACAP receptors, though which the peptide exerts its neurotrophic and neuroprotective potency; one such destination being glial cells, which are involved in neural communication: another element attenuated in the schizophrenic brain. Recent studies on humans reported that genetic variants of the PACAP and PAC1 genes are associated with schizophrenia and that the risk of single nucleotide polymorphism (SNP) of these genes could be associated with reduced hippocampal volume and poorer memory performance [19].

Studies indicate that disturbances of PACAP function might be associated with schizophrenia, and PACAP knockout mice (PACAP^−/−^ mice) could be a possible animal model for examining schizophrenia. PAC1 receptor-deficient mice have also exhibited abnormal behavior [41]. Interestingly, PAC1-overexpression mice have been reported to display strikingly similar phenotypes to PAC1 knockout mice [42], suggesting that the changes in the expression of PACAPergic signaling observed in schizophrenic patients seem to be very complex, and associated with defects or overexpression of PAC1. The conventional drugs which are used to treat schizophrenia or potential candidates may restore the normal conditions for PACAP-PAC1 signaling in the brain, perhaps by acting like partial agonists [43].

Our findings indicate that 24-h incubation with 20 µM haloperidol and olanzapine caused a statistically significant reduction of PAC1 mRNA expression; however, the effect was not maintained after prolonged treatment with drugs, and haloperidol even up-regulated PAC1 mRNA expression in a dose-dependent manner. It was found that 5 µM haloperidol administration resulted in 1.8-fold greater PAC1 mRNA expression than controls, while 20 µM haloperidol was associated with a 2.5-fold increase. The influence of amisulpride on PAC1 mRNA was not statistically significant.

Longer incubation times with haloperidol or olanzapine were associated with the opposite effect on PAC1 gene expression; as neuroleptics are used for longer than 24-h periods, the obtained results concerning 72-h incubation with drugs should be taken into consideration. Although the results from in vitro studies cannot be easily transferred to in vivo studies, it is possible that the effect observed in the cell cultures exposed to neuroleptic drugs for 72 h may be related to the delayed effect observed in the animal and clinical studies. It seems to be consistent with findings that the mechanisms by which neuroleptics produce their therapeutic effects (seen after a few weeks of continuous treatment) must involve the chronic regulation of various second messenger systems following the immediate receptor blockade [8]. Therefore, it is possible that the increase of mRNA PAC1 expression, i.e. the first step of the PACAPergic pathway, by haloperidol and olanzapine may influence the PACAP/PAC1 receptor system in glial cells, e.g. activation of cAMP/protein kinase A (PKA)-dependent signaling pathways, which are important in appropriate astrocyte function (differentiation, maturation, glutamate turnover or antioxidant protection) [44, 45]. Disturbances of these regulatory mechanisms in glial cells could represent a major cause for neurological and psychiatric disorders [1]. Therefore, the positive modulation of cAMP signaling may promote the normal state of differentiated astrocytes and favor the protection and function of neuronal networks.

A strong genetic link exists between schizophrenia and VPAC2 receptors: two recent publications from independent groups found that schizophrenia was associated with the presence of an increased number of the gene copies encoding the VPAC2 receptor, caused by duplications of the 7q36.3 chromosomal region VIPR2 [20, 21]. This copy number increase was associated with the elevated gene expression and activity of VPAC2 in cells lines carrying the duplication. Such a selective antagonist of VPAC2 receptors could offer therapeutic potential in the treatment of patients who carry duplication of the VPAC2 region. Our findings indicate that 20 µM haloperidol, 20 µM olanzapine and 100 µM amisulpride decreased VPAC2 mRNA expression, especially after 72-h incubation, with haloperidol being distinctly more potent than atypical neuroleptic drugs. A 24-h incubation with 20 µM haloperidol resulted in 20% decreased VPAC2 mRNA expression with the effect reduced by 65% after prolonged treatment. Olanzapine decreased the VPAC2 expression by approximately 30%, and amisulpride by 16%. To date, no studies have been conducted to demonstrate the relationship between schizophrenia and VPAC1 receptors.

The literature data indicates that the perfect neuroleptic drug should inhibit VPAC2 mRNA expression and restore the appropriate balance of PAC1 receptor expression in different regions of the brain [20, 21, 41, 42]. The tested drugs seem to meet these criteria, at least when it comes to the VPAC2 receptors, which are overexpressed in schizophrenia. All of the tested drugs inhibited VPAC2 after long-term treatment, albeit limited by in vitro studies, and this effect could be a direction for future neuroleptics to take regarding their therapeutic activity. An opposite effect was observed concerning PAC1 receptor expression: after 72-h incubation, haloperidol and olanzapine increased the mRNA level of PAC1 receptors, but amisulpride slightly inhibited expression at a lower concentration and had no effect at a higher concentration. Haloperidol, a typical neuroleptic, was more potent in increasing PAC1 mRNA expression and inhibiting VPAC2 mRNA expression than the tested atypical neuroleptics. The diverse potency of typical and atypical antipsychotics on the mRNA level of PAC1 and VPAC2 receptors may explain the differences in their therapeutic efficacy used for treating patients with schizophrenia.

Moreover our findings indicate that glial cells may be a possible target site of action of neuroleptics. This hypothesis is particularly important when considering that glial cells are more numerous than nerve cells in the brain, and abnormal neuronal network formation and neural transmission are common in schizophrenia [1, 2]. PACAP is involved in both neuronal transmission and neuronal development, suggesting that the regulation of the PACAP system through glial cells might be one of the strategies for the treatment of schizophrenia [10].

The study also examined the effect of exogenous PACAP38 on the expression of its specific receptors as reference to the neuroleptic drugs. A slight up-regulation of PAC1 mRNA was observed after a 24-h incubation with PACAP38. This effect was also noted after a further 72 h of treatment with peptide. A similar situation was noticed for VPAC2 protein level: PACAP38 incubation produced a significant six-fold increase of VPAC2 mRNA expression after a 24-h incubation, although this effect was weaker after 72 h. The molecular basis of the observed positive feedback mechanism can be explained by the fact that PACAP as a neuropeptide with pleiotropic potency by increasing its specific receptors enhances PACAP signaling system. The obtained results correspond with previous studies, which note that PACAP increased PAC1 receptor expression in LβT2 gonadotroph cells [46]. Moreover, PACAP stimulates the expression of its own mRNA in PC12 cells as well as cultured rat cortical neurons [47]. It has been suggested that the expression of PACAP and its receptors may be upregulated by PACAP via an autocrine mechanism, and that the role of endogenous PACAP is to promote the survival of nerve cells [48, 49]. It is worth noting that, similar to exogenous PACAP38, the neuroleptic drugs tested in the present study also increased PAC1 mRNA expression, which can contribute to a neuroprotective mechanism of action. This finding correlates with recent observations that antipsychotics not only act on dopaminergic and serotoninergic neurotransmission but with prolong treatment may also influence neurotrophic and other growth-related factors [7, 8, 25].

The study does have some limitations. First, the expression of the genes was tested in only one cell line, glioblastoma T98G, which may be regarded as of limited relevance to the brain pathology observed in schizophrenia. Second, changes in mRNA levels do not necessarily mean alterations in protein levels. Follow-up experiments to measure the expression of PAC1 and VPAC2 protein levels are needed to provide supporting data.

In conclusion, the present work provides the first confirmation of the influence of neuroleptic drugs on the expression of genes encoding the PAC1/VPAC type receptors in T98G cells. The tested antipsychotics differentially regulated the changes in PACAP receptor levels depending on the concentration and incubation time: While all drugs inhibited VPAC2 mRNA expression after long-term treatment, only haloperidol and olanzapine up-regulated PAC1 receptor mRNA expression. Our findings offer a completely new perspective on the neuroprotective mechanism of action of antipsychotics and open new opportunities for novel therapeutic targets. Although antipsychotics are the best treatment of schizophrenia currently available, they have many side effects, such as extrapyramidal side effects. A new strategy to avoid these side effects may be by exploiting the regulation of PACAPergic pathway. A key advantage of PACAP therapy is that it enhances not only dopaminergic and serotonergic transmission, but also neuronal activity, a mechanism which may improve efficacy to a greater degree than traditional treatment strategies; a quality which is the cornerstone of effective schizophrenia therapy.
